# Clinical and serological tests for arboviruses in free-living domestic pigeons (*Columba livia*)

**DOI:** 10.1590/0074-02760170014

**Published:** 2017-08

**Authors:** Bruna Alves Ramos, Jannifer Oliveira Chiang, Lívia Carício Martins, Liliane Leal das Chagas, Franko de Arruda e Silva, Milene Silveira Ferreira, Maria Nazaré Oliveira Freitas, Bianca Nascimento de Alcantara, Sandro Patroca da Silva, Stefânia Araújo Miranda, Barbara Alves Sepulvreda, Layna Thayssa Guimarães Corrêa, Andréa Maria Góes Negrão, Pedro Fernando da Costa Vasconcelos, Alexandre do Rosário Casseb

**Affiliations:** 1 Universidade Federal do Pará, Instituto de Ciências Biológicas, Belém, PA, Brasil; 2 Instituto Evandro Chagas, Setor de Arbovirologia e Febres Hemorrágicas, Ananindeua, Pará, Brasil; 3 Parque Naturalístico Mangal das Garças, Setor Veterinário, Belém, PA, Brasil; 4 Universidade Federal Rural da Amazônia, Instituto da Saúde e Produção Animal, Laboratório de Biologia Molecular, Belém, PA, Brasil

**Keywords:** arbovirus, domestic pigeons, clinical, serological tests

## Abstract

**BACKGROUND:**

In this study, we evaluated the role of free-living domestic pigeons (*Columba livia*) as a reservoir of arboviruses in the city of Belém, state of Pará, Brazil. We investigated the presence of antibodies against the most prevalent arboviruses.

**OBJECTIVES:**

This study was aimed at evaluating some clinical and physical parameters of domestic pigeons, including the presence of antibodies to Amazon-endemic arboviruses.

**METHODS:**

Eighty-five healthy pigeons were captured in Mangal das Garças Park, in Belém, and were bled. Upon capture, the birds were subjected to a clinical examination in search of alterations that could indicate the presence of arboviruses. Blood samples were converted to serum and tested using the haemagglutination inhibition (HI) technique with a panel of 19 antigens of arboviruses circulating in the Amazon. The confirmation assay for the positive reactions to the viral species tested by HI was a neutralisation test in new-born Swiss albino mice (*Mus musculus*) [mouse neutralisation test (MNT)].

**FINDINGS:**

A total of 10 (11.8%) serum samples tested positive for antiflavivirus antibodies by HI. All the samples positive for the HI test were subjected to MNT for detection of viruses and yielded negative results (logarithmic neutralisation index < 1.7).

**MAIN CONCLUSION:**

The results represent the first serological detection of antiarbovirus antibodies in domestic pigeons as potential hosts of arboviruses in Brazil. The detection of haemagglutination-inhibiting antibodies against genus Flavivirus indicated that there was recent contact between the analysed domestic pigeons and these arboviruses. Further studies are needed to evaluate the role of free-living pigeons in the maintenance cycle and spread of arboviruses in the Amazon.

In Brazil, the Amazon rainforest is considered the most important arbovirus ecosystem because of its favourable climatic and environmental conditions; indeed, in this rich ecosystem, a wide variety of wild animals and bloodsucking arthropods share the ecologic niche (Casseb et al. 2013, Vasconcelos et al. 2001). Approximately 212 viral species have been isolated in the Brazilian Amazon; 104 of these are unique to the region and 36 are associated with infections in humans [International Committee on Taxonomy of Viruses (ICTV) 2015]. Several bloodsucking arthropods may be associated with the transmission of arboviruses. In fact, mosquitoes, sandflies, ticks, and midges participate in the maintenance cycle of several known arboviruses (Henriques 2008).

Birds occupy the third position as natural sources of arboviruses. Various avian species are considered primary hosts of arboviruses, acting as natural amplifiers and serving as a source of infection for vectors (Araújo et al. 2012).

Domestic pigeons (*Columba livia*, Aves: Columbidae) are synanthropic birds descending from rock pigeons, wild Columbidae from Asia, Europe, and North Africa that inhabit plateaus, cliffs, and slopes. They were introduced into Brazil by Europeans, in the XIV century, during the colonisation, and are well adapted to urban centres and rural areas, because of the abundant availability of food and shelter and the absence of natural predators (Nunes & Miranda 2010).

The direct interaction between domestic pigeons and humans and other animals, for example, in urban or rural areas, is not beneficial, because these birds are hosts or reservoirs of ~60 types of infectious agents, from bacteria to fungi, viruses, protozoa, and parasites with a zoonotic potential, and are a cause for concern in terms of public and animal health (Nunes 2003).

In cities, there is a large population of domestic pigeons in public places (squares, streets, fairs, and ecological parks), owing to the abundance of food available in the trash or provided directly by the people and shelter. This situation predetermines the emergence of zoonotic diseases such as salmonellosis, mycoplasmosis, histoplasmosis, cryptococcosis, Newcastle disease, and the wild cycle of arboviruses, such as West Nile virus (WNV), Saint Louis encephalitis virus (SLEV), and Eastern equine encephalomyelitis virus (EEEV) (Gruwell et al. 2000, Komar 2003, Nunes 2003).

No study has been carried out in Brazil to assess the potential of domestic pigeons as reservoirs of arboviruses. Thus, this study was aimed at evaluating some clinical parameters of domestic pigeons, namely, the presence of antibodies to Amazon endemic arboviruses.

## MATERIALS AND METHODS

All the procedures described below were carried out with the consent of the Ethical Committee for Animal Use (CEUA) of the Federal Rural University of Amazonia (UFRA) and Biodiversity Authorization and Information System (Sisbio)/Brazilian Institute of Environment and Renewable Natural Resources (Ibama) under registrations 23084.007559/2016-59 (UFRA) and 69765274, respectively.

**Fig. 1:**
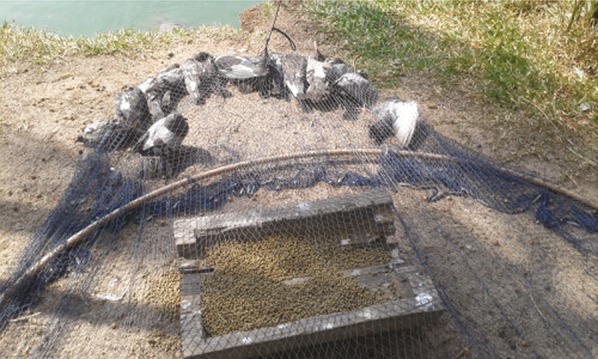
: domestic pigeons (*Columba livia*) captured in the Mangal das Garças Natural Park, Belém, state of Pará, trapped in a circular net with borders made of a polyvinyl chloride pipe.

*Capture of birds* – Eighty-five domestic pigeons (*C. livia*) were captured in the Mangal das Garças Natural Park (PNMG), in the city of Belém, state of Pará (PA), from January to October 2015. A trap (Fig. 1), made of a circular net attached on its borders to a polyvinyl chloride pipe rim, was used for the capture.

*Clinical evaluation* – Upon capture, the birds were subjected to a clinical examination in search of alterations that could indicate the presence of arboviruses. Data such as gender, age (young or adult), body condition [“good, fair, and bad” scores, according to the scheme described by Fontenelle and Barros (2014)], the general condition (a smart animal, responsive to stimuli: classified as good; an apathetic animal, prostrated, or cachectic: classified as bad), the plumage (according to appearance, classifications: good or bad), the presence or absence of ectoparasites, and the status of mucous membranes (coloration, capillary perfusion test, and absence of lesions, classification: normal or altered) were analysed.

*Obtaining serum samples* – For blood collection, the animals were anesthetised beforehand using a combination of ketamine hydrochloride (30 mg/kg) and xylazine hydrochloride (6 mg/kg), according to the protocol described by Altman et al. (1997). Blood (10 mL) was collected via the intracardiac route accessed from the cranial region. The samples collected were centrifuged (2,000 rpm/10 min) to separate serum samples, which were then aspirated and stored in cryogenic microtubes at -70°C until analysis.

*Serological tests* – All the 85 serum samples obtained were subjected to a haemagglutination inhibition (HI) test according to the microplate technique described by Shope (1963), with a titration cut-off point of 20. They were tested for antibodies against 19 arboviruses present in the Brazilian Amazon, isolated by the Arbovirology and Hemorrhagic Fever Section (SAARB) of the Evandro Chagas Institute (IEC). This list includes members of the following viral families: Togaviridae (genus Alphavirus): EEEV, Western equine encephalomyelitis virus (WEEV), Mayaro virus (MAYV), Mucambo virus (MUCV); Flaviviridae (genus Flavivirus): SLEV, WNV, yellow fever virus (YFV), Ilheus virus (ILHV), Cacipacore virus (CPCV), Bussuquara virus (BSQV), Rocio virus (ROCV); Bunyaviridae (Orthobunyavirus, Phlebovirus, and non-grouped genera): Tacaiuma virus (TCMV), Maguari virus (MAGV), Utinga virus (UTIV), Caraparu virus (CARV), Oropouche virus (OROV), Catu virus (CATUV), Icoaraci virus (ICOV), and Belem virus (BLMV). The samples were also tested for antibodies against dengue virus (DENV) serotypes 1–4) and Zika virus (ZIKV), which are two Flaviviruses endemic to humans in Brazil.

For the HI test, serum samples of domestic pigeons pretreated with acetone P. A. for the removal of non-specific inhibitors were initially subjected to the screening step, where they were added into a 96-well microtitre plate (“U” shape wells) together with a viral suspension (4 HAU) and tested using a white goose (*Anser cinereus*) erythrocyte suspension diluted in dextrose, gelatine, and barbital; dilution 1:5 (DGV). The samples positive at the screening step were diluted with bovine serum albumin (0.4%) up to the ratio of 1/1,280 to obtain the titre of the antibodies present in the serum samples.

The confirmation assay for the positive reactions to the viral species tested by the HI method was a neutralisation test in new-born Swiss albino mice (*Mus musculus*) [mouse neutralisation test (MNT)], according to the technique described by Casals (1967). For this test, the serum samples of domestic pigeons that were antiarbovirus antibody-positive in the HI test were serially diluted in bovine albumin (0.4%) and inoculated intracerebrally into two-day-old Swiss albino mice. These animals were then monitored for a period of 21 days to assess the presence/absence of any signs of a neurological infection. Samples with a logarithmic neutralisation index (LNI) greater than or equal to 1.7 [half-lethal dose (LD_50_)/0.02 mL) were considered positive based on the mortality and survival index observed in the mouse cages during the MNT period.

## RESULTS

*Clinical evaluation* – Of the 85 birds captured, 44 (51.8%) were males and 41 (48.2%) were females. Among the males, 21 (47.7%) were young and 23 (52.3%) were adults; among the females, 23 (56.1%) were young and 18 (43.9%) were adults. During the evaluation of the clinical parameters, 15 birds (17.6%) were found to have a good body condition, 29 (34.1%) got a fair score, and 41 (48.3%) received a bad score. With regard to the general condition, 84 pigeons (98.8%) were classified as good and only one (1.2%) was classified as bad. Regarding the mucosa, 51 (60%) birds had ocular mucosa within the normal range and, in 34 birds (40%), it was altered; 68 (80%) had the oral mucosa without alterations and 17 (20%) had an altered mucosa, 60 (70.6%) had an unchanged nasal mucosa, which was altered in 25 (29.4%) birds; 76 (89.4%) had the cloacal mucosa without alterations and nine (10.6%) showed alterations. As for plumage, 55 birds (64.7%) had beautiful and healthy feathers, receiving a good rating, whereas 30 (35.3%) had ugly and weak feathers and were, therefore, rated as bad. The presence of ectoparasites was observed in all the evaluated birds; among the captured birds, 17 (20%) had only chewing lice, six (7.1%) had only haematophagous flies (genus *Pseudolynchia* spp), and 68 (72.9%) had both lice and flies.

**Fig. 2:**
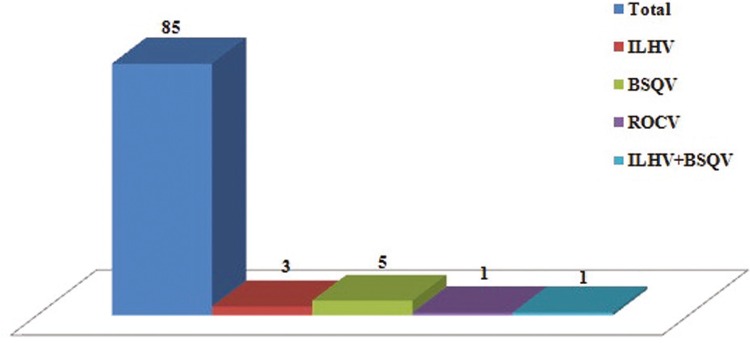
: monotypic and heterotypic reactions for Flavivirus observed in serum samples of domestic pigeons (*Columba livia*) during the haemagglutination inhibition test. BSQV: Bussuquara virus; ILHV: Ilheus virus; ROCV: Rocio virus.

*Serological tests* – Of the 85 serum samples tested for antibodies in the present study, 10 showed a positive reaction to ILHV, BSQV, and ROCV (Flaviviridae family), three showed monotypic reactions (reaction to only one type of arbovirus) for ILHV, five for BSQV, one for ROCV, and one showed a heterotypic reaction (reaction to more than one arbovirus) to ILHV and BSQV viruses, respectively, according to Figure 2. All the serum samples were tested for DENV and ZIKV, but there were no positive results. The 10 samples positive for the HI test were subjected to MNT for detection of viruses, but all the samples yielded a negative result (LNI < 1.7).

## DISCUSSION

In general, none of the birds tested in the present study showed clinical signs of arbovirus infection at the time of the clinical examination, such as neurological and/or haemorrhagic lesions. According to Ferreira (2012), domestic pigeons are resistant to most of the zoonotic microorganisms they carry, presenting asympto- matic infections and silently disseminating the etiological agents to humans and animals.

All the evaluated pigeons carried ectoparasites and most of the birds were parasitised by an association between chewing lice and haematophagous flies. Cezar (2005) mentioned that some genera of chewing lice of the family Menoponidae are common parasites of domestic pigeons and feed on the barbs of their feathers. As a result, they can modify the appearance and cause a lack of vitality and promote falling out of feathers and deformities in the plumage. In this study, 27 (31.8%) of the evaluated birds that got a bad score on the plumage were parasitised by chewing lice.

According to Amaral et al. (2012), in Brazil, there are reports of two species of haematophagous flies present on domestic pigeons: *Pseudolynchia canariensis* and *Pseudolynchia brunei*. Marques et al. (2010) reported the presence of bacteria and fungi as well as eggs of certain endoparasites in the legs and buccal apparatus of this species of flies, indicating a possible role of these insects in the mechanical deposition of infectious and parasitic agents onto domestic pigeons.

Even though they are considered specific parasites of Columbidae, Gredilha et al. (2008) mentioned the presence of these flies in Falconiformes in the state of Rio de Janeiro, indicating the possibility of adaptation of this insect to new avian hosts. There are no studies showing any involvement of *Pseudolynchia* spp in the transmission of diseases to humans and other animals or their potential as vectors in the cycle of arboviruses that may infect pigeons and other avian species.

In this study, all 10 serum samples positive for antiflavivirus antibodies in the HI analysis tested negative in the MNT in mice. Although it is considered extremely effective at the detection of arbovirus antibodies in avian biological samples owing to its low cost and ease of execution, the HI test has a high rate of cross-reactivity, mainly in relation to viruses belonging to the same family. In the present study, only one sample of domestic-pigeon serum showed a cross-reaction between flaviviruses ILHV and BSQV. According to Araújo et al. (2009), the HI test is sufficiently sensitive for the detection of antibodies, but less specific than MNT, and is prone to cross-reactions, which are mainly observed for viruses of genus Flavivirus. According to Arruda and Fonseca (2015), antibodies that do not exhibit neutralizing activity (such as haemagglutination-inhibitory antibodies) can be produced by B lymphocytes through recognition of fragments or external or internal viral proteins. Diamond (2003) mentioned that antibodies produced during infections by viruses of genus Flavivirus recognise structural proteins M and E because they are common among several species of Flavivirus. This situation leads to cross-reactions in tests with lower specificity such as HI. Most of the monotypic positive reactions visualised for flaviviruses ILHV, BSQV, and ROCV in the HI test showed antibody titres equal to 20, coinciding with the cut-off established for this test. According to Gibbs et al. (2005), the presence of haemagglutination-inhibiting antibodies to an arbovirus in serum of domestic pigeons indicates contact or recent infection; these antibodies are less durable than the neutralizing antibodies, which are produced late in this species of bird.

Holmes and Twiddy (2003) and Lanciotti et al. (2008) reported that both DENV and ZIKV are arboviruses of African origin and initially had a wild cycle involving non-human primates and mosquitoes of genus *Aedes*. Currently, both viruses are found to be circulating in rural and urban areas, in cycles involving mosquitoes of species *Aedes aegypti* and humans. Although they are synanthropic and widely distributed in rural and urban areas, there are no reports on the involvement of domestic pigeons in the maintenance and transmission cycle of DENV (serotypes 1–4) and ZIKV. This notion is supported by the present study because of the negative results obtained in the serological tests performed.

Although their role in the arbovirus transmission cycle towards humans and other animals has not been fully elucidated, domestic pigeons can, nevertheless, act as sentinel hosts during outbreaks involving arboviruses. They become infected with a disease and develop detectable antibody titres or die suddenly; both situations are indicative of arbovirus circulation. Epidemiological studies conducted in Florida by Blackmore et al. (2003), in New York by Komar (2003), and in Macedonia by Chaintoutis et al. (2014), demonstrate that serum samples collected from domestic pigeons can be used to monitor the circulation of WNV and other arboviruses during epidemic periods, both in urban and rural areas, and this avian species may be used in epidemiological surveys involving health surveillance.

Thus, the results of this study demonstrated the following: pigeons serologically positive for antiarbovirus antibodies may not necessarily show clinical signs of infection by these viruses; most birds that were antiarbovirus antibody-positive according to the HI test were parasitised by *Pseudolynchia* spp: haematophagous arthropods with an unknown potential for arbovirus transmission to animals and humans. The detection of haemagglutination-inhibiting antibodies against genus Flavivirus indicated that there was recent contact between the analysed domestic pigeons and these arboviruses; the birds under study did not show the presence of antibodies to the DENV and ZIKV viruses. Being the first report describing the presence of antibodies against zoonotic arboviruses in serum samples of domestic pigeons in Brazil, this study has significant relevance to public health because pigeons are synanthropic birds that live directly with humans and animals in urban and rural areas.
